# Association of glycemic variability with death and severe consciousness disturbance among critically ill patients with cerebrovascular disease: analysis of the MIMIC-IV database

**DOI:** 10.1186/s12933-023-02048-3

**Published:** 2023-11-16

**Authors:** Weimin Cai, Yaling Li, Kun Guo, Xiao Wu, Chao Chen, Xinran Lin

**Affiliations:** 1 Department of Neurology, The First Affiliated Hospital of Wenzhou Medical University, Wenzhou, 325000 China; 2 Department Health Management Center, the Second Affiliated Hospital, School of Medicine, Zhejiang University, Hangzhou, 31000 China; 3 Department of Gastroenterology and Hepatology, The First Affiliated Hospital of Wenzhou Medical University, No. 2, Fuxue Lane, Wenzhou, 325000 China

**Keywords:** Glycemic variability, Cerebral infarction, Non-traumatic cerebral hemorrhage, ICU, Blood glucose

## Abstract

**Background:**

The association of glycemic variability with severe consciousness disturbance and in-hospital all-cause mortality in critically ill patients with cerebrovascular disease (CVD) remains unclear, This study aimed to investigate the association of glycemic variability with cognitive impairment and in-hospital death.

**Method:**

We extracted all blood glucose measurements of patients diagnosed with CVD from the Medical Information Mart for Intensive Care IV (MIMIC-IV). Glycemic variability was defined as the coefficient of variation (CV), which was determined using the ratio of standard deviation and the mean blood glucose levels. Cox hazard regression models were applied to analyze the link between glycemic variability and outcomes. We also analyzed non-linear relationship between outcome indicators and glycemic variability using restricted cubic spline curves.

**Results:**

The present study included 2967 patients diagnosed with cerebral infarction and 1842 patients diagnosed with non-traumatic cerebral hemorrhage. Log-transformed CV was significantly related to cognitive impairment and in-hospital mortality, as determined by Cox regression. Increasing log-transformed CV was approximately linearly with the risk of cognitive impairment and in-hospital mortality.

**Conclusion:**

High glycemic variability was found to be an independent risk factor for severe cognitive decline and in-hospital mortality in critically ill patients with CVD. Our study indicated that enhancing stability of glycemic variability may reduced adverse outcomes in patients with severe CVD.

**Supplementary Information:**

The online version contains supplementary material available at 10.1186/s12933-023-02048-3.

## Introduction

Cerebrovascular disease (CVD) remains one of the major global health issues, and exerts substantial economic burden on patients and society [1]. Incident CVD is associated with accelerated, persistent cognitive impairment, poorer quality of life and higher risk of death [2, 3]. Patients with CVD admitted to an intensive care unit (ICU) are more likely to have severe cognitive impairment, more complex disease condition, and an increased mortality rate [4, 5].

Recently, growing evidence indicate that intensive insulin therapy increase the risk of adverse prognosis, in part due to blood glucose fluctuation and hypoglycemic events [6, 7]. Thus, high glycemic variability has been introduced to be an adverse prognostic indicator [8, 9]. Glycemic variability is measured by the fluctuation of blood glucose within a certain period, which has been regarded as the main pattern of abnormal blood glucose levels in critically ill patients [10]. Several risk factors contribute to unstable blood glucose levels, including stress hyperglycemia, advanced age, and medications (such as insulin, adrenaline and steriods) [11, 12]. Significant glycemic variability is related to an elevated risk of cardiovascular disease, CVD, and microvascular relevant disease [13–15], as well as endothelial cell damage, insulin resistance, inflammation [16–18]. Some pathophysiological processes were proposed to elucidate the possible effects of glycemic variability on brain. Glycemic levels are associated with amyloid burden, cause cognitive impairment in apolipoprotein E ε4 allele carriers [19, 20]. At the cellular level, significant glycemic variability has been shown to cause more endothelial dysfunction and induce oxidative stress than stable hyperglycemia [21], potentially contributing to more serious cerebrovascular impairment and cognitive decline. It has also been reported that oxidative stress, hyperglycemia, hypoglycemia, and other possible risk factors play important roles in cognitive decline [22], but the role of glycemic variability has received less attention, and its usefulness in clinical practice remains controversial [23, 24]. Therefore, we conducted this study to explore the relationship among severe cognitive impairment, glycemic variability and in-hospital death in critically ill patients with non-traumatic cerebral hemorrhage and cerebral infarction. This may help physicians identify patients at higher risk for closer monitoring or timely therapy.

## Method

### Study population

The present study was a retrospective analysis of the Medical Information Mart for Intensive Care IV (MIMIC-IV) database. MIMIC-IV is a publicly available database containing medical information on 76,943 ICU admissions for 53,150 unique patients to intensive critical care unit at Beth Israel Deaconess Medical Center (BIDMC) in Boston from 2008 to 2019 [25]. An approved researcher (Xinran, Lin) was responsible for data extraction.

Inclusion criteria: Patients diagnosed with non-traumatic hemorrhage or cerebral infarction according to the ICD-9 or ICD-10. Exclusion criteria: (a) not admitted to any ICU, (b) patients with a length of 6 h, (c) patients with fewer than three blood glucose measurements. (d) severe cognitive impairment prior to hospital admission (Glasgow coma scale score < 8). For patients with repeated admissions, we only collected the first admission relevant information.

### Patient characteristics

Structured Query Language (SQL) was applied to extract the relevant medical information from the MIMIC-IV database. The following data was obtained: (1) demographic information: age sex, race, body mass index (BMI); (2) comorbidities were determined based on ICD-9 or ICD-10, including coronary artery disease (CAD), chronic obstructive pulmonary disease (COPD), diabetes mellitus, heart failure, hypertension, sepsis, and atrial fibrillation (AF); (3) laboratory indicators included white blood cell (WBC), red blood cell (RBC), prothrombin time (PT), platelet, creatine, active partial thromboplastin time (PTT). (4) severity of disease included Acute Physiology Score III (APSIII), Oxford Acute Severity of Illness Score (OASIS), Simplified Acute Physiology Score (SAPSII), Sequential Organ Failure Assessment Score (SOFA), and Glasgow coma scale (GCS) score (5) Therapy: continuous renal replacement therapy (CRRT), mechanical ventilation (MV), antidiabetes therapy, and antihypertensive therapy.

### Exposure

Glycemic variation was based on blood glucose measurement records during the hospital stay. Blood glucose records were obtained real-time during clinical care. Due to the real-world nature of blood glucose being recorded varying by different patients, the frequency of blood glucose was measured on a case-dependent basis and the timing of patients’ measurement was not standardized. The coefficient of variation (CV) was used to describe glycemic variability, which is the ratio of the SD and the mean of all multiple measurements. Moreover, the glycemic variability was calculated based on the blood glucose information preceded the outcomes occurrence of the included patients.

### Outcome measures

Our primary endpoint was the occurrence of severe decline of consciousness, determined as a GCS score of less than eight within 30 days of patient admission. The secondary outcome measure was the in-hospital mortality within 30 days. In-hospital death was determined by the date of death and discharge, including deaths in the ICU and after transfer out of the ICU.

### Statistical analyses

Continuous variables were presented as the mean ± SD or median and interquartile range (IQR). The comparison of continuous variables was performed using t-test or ANOVA or using Mann–Whitney U-test or Kruskal–Wallis test, as appropriate. Categorical variables were expressed as numbers or percentages (%), and differences were compared among groups using the Pearson chi-square test or Fisher’s exact test.

Due to the non-normal distribution of CV, CV was natural log-transformed for analysis as a continuous variable, and stratified by tertiles of CV. Clinically relevant and prognosis-associated variables were enrolled in the multivariate model as confounding variables. Cox Hazard regression models were applied to evaluate hazard ratios (HRs) and their 95% confidence intervals (95% CIs) and adjusted for several confounding variables (Model 1: unadjusted; Model 2: adjusted for age, sex, race, and BMI; Model 3: adjusted for age, sex, race, BMI, CAD, diabetes, heart failure, hypertension, sepsis, AF, MV, CRRT, antidiabetes therapy, antihypertensive therapy, and glucose measurement number.). Additionally, a four-knots (5th, 35th, 65th, and 95th percentiles) restricted cubic spline (RCS) was used to present potential non-linear relationship between glycemic variability and outcomes. The Wald test was used to assess the presence of non-linearity. Pearson correlation coefficient was used to determine the relationship between glycemic variability and number of glycemic measurements and length of hospital stay. Sensitivity analyses were performed to determine the relationship between glycemic and occurrence of outcome in different times. Finally, subgroup analyses were conducted according to the prespecified subgroups, including age (> 65 or ≤ 65 years), BMI (BMI ≤ 30 kg/m^2^ or > 30 kg/m^2^), sex, CAD, diabetes, heart failure, hypertension, sepsis, and AF. All statistical analyses were performed using R statistical package (R version 4.2.2), and 2-side P < 0.05 was considered statistically significant.

## Results

### Cohort characteristics

**Fig. 1:**
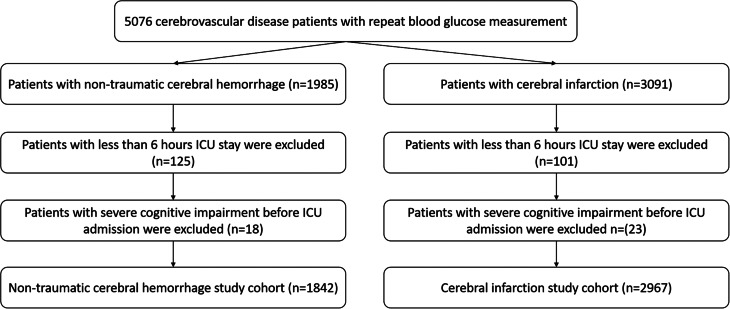
Flow of included patients through the trial

As shown in the Fig. 1, a total of 4809 patients were enrolled in this study (2967 patients diagnosed with cerebral infarction and 1842 patients diagnosed with non-traumatic cerebral hemorrhage). The median age of the non-traumatic cerebral hemorrhage was 67.72 years. There were 1018 males and 824 females with non-traumatic cerebral hemorrhage, of which 406 developed severe disturbance of consciousness and 322 died during the hospital stay (Table 1). There were 1549 men and 1418 women with cerebral infarction, of which 568 experienced severe disturbance of consciousness, and 469 died in the hospital (Table 2). Patients with non-traumatic cerebral hemorrhage and patients with cerebral infarction were stratified into three groups, respectively, based on the tertiles of CV (Non-traumatic cerebral hemorrhage: T1: CV < 14.1%, T2: CV 14.1–21.5%,T3: CV > 21.5%; Cerebral infarction: T1: CV < 16.0%, T2 CV 16.0-24.7%,T3: CV > 24.7%). The baseline characteristics were described based on the CV tertiles (Tables 1 and 2).

**Table 1:** Baseline characteristics of patients with non-traumatic cerebral hemorrhage according to tertiles of glycemic variability

Characteristics	Total patients n = 1842	T1 (< 14.1%) n = 614	T2 (14.1–21.5%) n = 614	T3 (> 21.5%) n = 614	p
Age	67.72 (55.54–79.24)	67.14 (54.51–79.71)	67.23 (54.57–78.88)	68.24 (58.09–78.82)	0.240
BMI	26.57 (23.03–30.9)	26.57 (22.89–30.85)	27.28 (23.5–31.52)	26.21 (22.87–30.46)	0.158
Sex					0.685
Female	824 (44.7)	278 (45.3)	266 (43.3)	280 (45.6)	
Male	1018 (55.3)	336 (54.7)	348 (56.7)	334 (54.4)	
Race (White)	1158 (62.9)	398 (64.8)	389 (63.3)	371 (60.4)	0.268
Systolic BP	128.47 (119–136.79)	129.44 (120.73–137.18)	128.71 (119.26–136.93)	127.07 (116.68–135.36)	0.012
Diastolic BP	65.33 (58.75–72.91)	66.47 (60.36–73.94)	65.52 (58.66–73.29)	63.84 (57.27–71.53)	< 0.001
Mean BP	83.16 (76.25–90.07)	84.16 (77.8–90.71)	83.55 (76.77–90.3)	81.75 (74.88–88.26)	< 0.001
Mean temperature	36.94 (36.7–37.25)	36.97 (36.74–37.25)	36.95 (36.73–37.28)	36.89 (36.65–37.24)	0.023
Mean glucose	128.78 (110.67–152.26)	119.67 (107.17–136.5)	126.67 (110–145.29)	147.88 (120.79–185.46)	< 0.001
CAD					< 0.001
No	1774 (96.3)	606 (98.6)	594 (96.7)	574 (93.5)	
Yes	68 (3.7)	8 (1.4)	20 (3.3)	40 (6.5)	
COPD					0.987
No	1760 (95.5)	586 (95.4)	587 (95.6)	587 (95.6)	
Yes	82 (4.5)	28 (4.6)	27 (4.4)	27 (4.4)	
Diabetes					< 0.001
No	1464 (79.5)	549 (89.4)	521 (84.9)	394 (64.2)	
Yes	378 (20.5)	65 (10.6)	93 (15.1)	220 (35.8)	
Heart failure					< 0.001
No	1665 (90.4)	571 (93.0)	561 (91.4)	533 (86.8)	
Yes	177 (9.6)	43 (7.0)	53 (8.6)	81 (13.2)	
Hypertension					0.249
No	775 (42.1)	250 (40.7)	250 (40.7)	275 (44.8)	
Yes	1067 (57.9)	364 (59.3)	364 (59.3)	339 (55.2)	
Sepsis					< 0.001
No	1730 (93.9)	602 (98.0)	580 (94.5)	548 (89.3)	
Yes	112 (6.1)	12 (2.0)	34 (5.5)	66 (10.7)	
AF					0.169
No	1475 (80.1)	489 (79.6)	506 (82.4)	480 (78.2)	
Yes	367 (19.9)	125 (20.4)	108 (17.6)	134 (21.8)	
SOFA	3 (2–5)	2 (1–4)	3 (2–4)	4 (2–6)	< 0.001
APSIII	34 (26–45)	30 (23–39)	33 (25–44)	40 (30–55.75)	< 0.001
SAPSII	32 (24–40)	29 (22–37)	31 (24–39)	36 (27–46)	< 0.001
OASIS	30 (24.25–36)	28 (23–33)	30 (25–36)	32 (26–38)	< 0.001
CCI	5 (3–7)	5 (3–6)	5 (3–7)	6 (4–8)	< 0.001
WBC	9.7 (7.4–12.6)	9.4 (7.6–11.7)	9.6 (7.4–12.3)	10.2 (7.3–14)	0.001
RBC	3.9 (3.36–4.39)	4.04 (3.57–4.44)	3.89 (3.33–4.4)	3.78 (3.22–4.3)	< 0.001
Platelet	214 (167–274)	212 (169.5–261.5)	222 (174–286)	206 (150.25–276.75)	0.001
PT	12.5 (11.6–13.6)	12.3 (11.5–13.2)	12.3 (11.5–13.5)	12.9 (11.8–14.3)	< 0.001
PTT	28.3 (25.8–31.5)	28.35 (25.7–31.1)	27.9 (25.9–30.78)	28.7 (25.8–32.2)	0.037
Creatine	0.8 (0.6–1.1)	0.8 (0.6–1)	0.8 (0.6–1)	0.9 (0.7–1.2)	< 0.001
Sodium	139 (137–142)	139 (137–142)	140 (137–142)	139 (137–142)	0.724
Potassium	3.9 (3.6–4.2)	3.9 (3.6–4.2)	3.9 (3.7–4.2)	3.9 (3.6–4.3)	0.882
Hemoglobin	11.9 (10.2–13.2)	12.4 (10.8–13.5)	11.8 (10.2–13.2)	11.4 (9.8–12.9)	< 0.001
MCH	30.5 (29.1–31.8)	30.6 (29.2–32)	30.5 (29.1–31.8)	30.5 (29.05–31.75)	0.192
MCHC	33.3 (32.4–34.3)	33.4 (32.5–34.42)	33.3 (32.3–34.3)	33.4 (32.3–34.3)	0.103
MCV	91 (87–95)	91 (88–95)	91 (87–95)	91 (87–95)	0.822
Antidiabetes therapy	1427 (77.5)	416 (67.8)	475 (77.4)	536 (87.3)	< 0.001
Antihypertensive therapy	1523 (82.7)	496 (80.8)	533 (86.8)	494 (80.5)	0.004
MV	716 (38.9)	165 (26.9)	250 (40.7)	301 (49.0)	< 0.001
CRRT	33 (1.8)	2 (0.3)	6 (0.9)	25 (4.1)	< 0.001
Hospital LOS	9.07 (5.49–15.41)	7.24 (4.82–11.67)	10.66 (6.79–17.74)	10.02 (5.25–17.9)	< 0.001
ICU LOS	3.78 (1.92–7.76)	3.28 (1.74–6.57)	4.61 (2.19–9.18)	3.75 (1.93–7.68)	< 0.001

Abbreviations: BMI, body mass index; BP, blood pressure; CAD, coronary artery disease; COPD, chronic obstructive pulmonary disease; AF, atrial fibrillation; SOFA, Sequential Organ Failure Assessment Score; OASIS, Oxford Acute Severity of Illness Score; SAPSII, Simplified Acute Physiology Score; CCI, Charlson comorbidity index; WBC, white blood cell; RBC, red blood cell; PT, prothrombin time; PTT, active partial thromboplastin time; MCH, mean corpuscular hemoglobin; MCHC, mean corpuscular hemoglobin concentration; MCV, Mean Corpuscular Volume; MV, mechanical ventilation; CRRT, continuous renal replacement therapy; LOS, length of stay; ICU, intensive care unit

**Table 2:** Baseline characteristics of patients with cerebral infarction according to tertiles of glycemic variability

Characteristics	Total patients n = 2967	T1 (< 16.0%) n = 989	T2 (16.0-24.7%) n = 989	T3 (> 24.7%) n = 989	p
Age	73.11 (62.13–82.53)	71.81 (60.25–82.04)	74.11 (63.36–83.06)	73.39 (63.59–82.49)	0.004
BMI	27.06 (23.38–31.33)	27.32 (23.38–31.84)	26.76 (23.44–30.82)	27.06 (23.28–31.45)	0.254
Sex					0.666
Female	1418 (47.8)	463 (46.8)	483 (48.8)	472 (47.7)	
Male	1549 (52.2)	526 (53.2)	506 (51.2)	517 (52.3)	
Race (White)	1923 (64.8)	646 (65.3)	638 (64.5)	639 (64.6)	0.921
Systolic BP	125.05 (112.85–140.58)	130.48 (116.5–144.63)	122.92 (111.89–138.34)	122.84 (110.78–137.01)	< 0.001
Diastolic BP	63.81 (55.78–73.14)	67.29 (58.72–76.28)	62.26 (54.84–72.30)	61.74 (54.75–70.35)	< 0.001
Mean BP	81.14 (73.20–90.64)	84.31 (75.87–94.02)	80.11 (72.39–90.04)	79.09 (71.89–88.00)	< 0.001
Mean temperature	36.85 (36.63–37.12)	36.89 (36.68–37.12)	36.82 (36.61–37.12)	36.83 (36.60–37.13)	< 0.001
Mean glucose	129.61 (112.25–157.43)	119 (105.60–135.45)	131 (115.00–154.08)	144.42 (121.20–187.67)	< 0.001
CAD					< 0.001
No	2460 (82.9)	870 (88.0)	809 (81.8)	781 (79.0)	
Yes	507 (17.1)	119 (12.0)	180 (18.2)	208 (21.0)	
COPD					0.078
No	2781 (93.7)	935 (94.5)	933 (94.3)	913 (92.3)	
Yes	186 (6.3)	54 (5.5)	56 (5.7)	76 (7.7)	
Diabetes					< 0.001
No	1916 (64.6)	769 (77.8)	669 (67.6)	478 (48.3)	
Yes	1051 (35.4)	220 (22.2)	320 (32.4)	511 (51.7)	
Heart failure					< 0.001
No	2169 (73.1)	820 (82.9)	702 (71.0)	647 (65.4)	
Yes	798 (26.9)	169 (17.1)	287 (29.0)	342 (34.6)	
Hypertension					0.002
No	1354 (45.6)	406 (41.1)	475 (48.0)	473 (47.8)	
Yes	1613 (54.4)	583 (58.9)	514 (52.0)	516 (52.2)	
Sepsis					< 0.001
No	2645 (89.1)	928 (93.8)	889 (89.9)	828 (83.7)	
Yes	322 (10.9)	61 (6.2)	100 (10.1)	161 (16.3)	
Atrial fibrillation					0.006
No	1812 (61.1)	640 (64.7)	571 (57.7)	601 (60.8)	
Yes	1155 (38.9)	349 (35.3)	418 (42.3)	388 (39.2)	
SOFA	4 (2–6)	3 (2–5)	4 (2–6)	4 (3–7)	< 0.001
APSIII	39 (29–52)	34 (26–44)	39 (29–52)	45 (33–60)	< 0.001
SAPSII	35 (28–43)	31 (25–39)	35 (29–42)	38 (30–47)	< 0.001
OASIS	31 (26–37)	30 (25–35)	32 (26–38)	32 (27–39)	< 0.001
CCI	7 (5–8)	6 (4–8)	7 (5–8)	7 (5–9)	< 0.001
WBC	9.7 (7.45–13)	9.4 (7.3–12.2)	10.1 (7.7–13.2)	9.9 (7.5–13.6)	0.001
RBC	3.67 (3.13–4.22)	3.87 (3.29–4.37)	3.63 (3.12–4.16)	3.54 (2.99–4.11)	< 0.001
Platelet	211 (158–280)	213 (165.5–273)	209 (158–280)	210 (149–286.5)	0.597
PT	13.1 (11.9–14.8)	12.7 (11.7–14.2)	13.1 (11.9–14.8)	13.4 (12.1–15.2)	< 0.001
PTT	29.8 (26.7–36)	29.3 (26.5–34.9)	29.95 (26.8–35)	30.1 (26.8–38)	0.068
Creatine	0.9 (0.7–1.3)	0.9 (0.7–1.1)	0.9 (0.7–1.3)	1 (0.7–1.4)	< 0.001
Sodium	140 (137–142)	140 (137–142)	139 (137–142)	140 (137–142)	0.151
Potassium	4 (3.7–4.4)	4 (3.7–4.3)	4 (3.8–4.4)	4.1 (3.8–4.5)	0.002
Hemoglobin	10.9 (9.3–12.6)	11.5 (9.8–13.1)	10.8 (9.4–12.5)	10.6 (8.9–12.2)	< 0.001
MCH	30.1 (28.7–31.4)	30.15 (28.8–31.4)	30.3 (28.8–31.6)	29.9 (28.5–31.2)	0.004
MCHC	32.9 (31.9–33.8)	33 (32.0–34.0)	33 (32.0–33.9)	32.8 (31.7–33.7)	< 0.001
MCV	91 (87–95)	91 (87–95)	91 (88–95)	91 (87–95)	0.209
Antidiabetes therapy	2353 (79.3)	697 (70.5)	811 (82.0)	845 (85.4)	< 0.001
Antihypertensive therapy	2405 (81.1)	745 (75.3)	840 (84.9)	820 (82.9)	< 0.001
MV	1230 (41.5)	316 (32.0)	447 (45.2)	467 (47.2)	< 0.001
CRRT	85 (2.9)	18 (1.8)	27 (2.7)	40 (4.0)	0.012
Hospital LOS	8.94 (5.67–15.5)	7.58 (4.95–12)	9.69 (6.06–16.65)	10.48 (6.09–17.16)	< 0.001
ICU LOS	3.08 (1.69–6.28)	2.71 (1.51–5.4)	3.19 (1.84–6.73)	3.3 (1.71–6.97)	< 0.001

Abbreviations: BMI, body mass index; BP, blood pressure; CAD, coronary artery disease; COPD, chronic obstructive pulmonary disease; AF, atrial fibrillation; SOFA, Sequential Organ Failure Assessment Score; OASIS, Oxford Acute Severity of Illness Score; SAPSII, Simplified Acute Physiology Score; CCI, Charlson comorbidity index; WBC, white blood cell; RBC, red blood cell; PT, prothrombin time; PTT, active partial thromboplastin time; MCH, mean corpuscular hemoglobin; MCHC, mean corpuscular hemoglobin concentration; MCV, Mean Corpuscular Volume; MV, mechanical ventilation; CRRT, continuous renal replacement therapy; LOS, length of stay; ICU, intensive care unit

Patients with higher CV levels were more likely to have comorbid diseases including CAD (p < 0.001), diabetes (p < 0.001), heart failure (p < 0.001), sepsis (p < 0.001), as well as higher disease severity score (SOFA, APSIII, SAPSII, OASIS, p < 0.001), higher levels of WBC (p < 0.001), PT (p < 0.001), creatine (p < 0.001) in patients with non-traumatic hemorrhage, and were more likely to have accepted antidiabetes therapy, antihypertensive therapy, mechanical ventilation, continuous renal replacement therapy (all p < 0.05) (Table 1).

Patients diagnosed with cerebral infarction exhibited similar results. Patients with higher CV values were more likely to combine CAD (p < 0.001), diabetes (p < 0.001), heart failure (p < 0.001), sepsis (p < 0.001), as well as higher disease severity scores (SOFA, APSIII, SAPSII, OASIS, p < 0.001), higher levels of PT (p < 0.001), creatine (p < 0.001), longer length of hospital and ICU stay (p < 0.001), and were more likely to have accepted antidiabetes therapy, antihypertensive therapy, mechanical ventilation, continuous renal replacement therapy (all p < 0.05) (Table 2).

### Glycemic variability was associated with severe disturbance of consciousness and in-hospital death

**Fig. 2:**
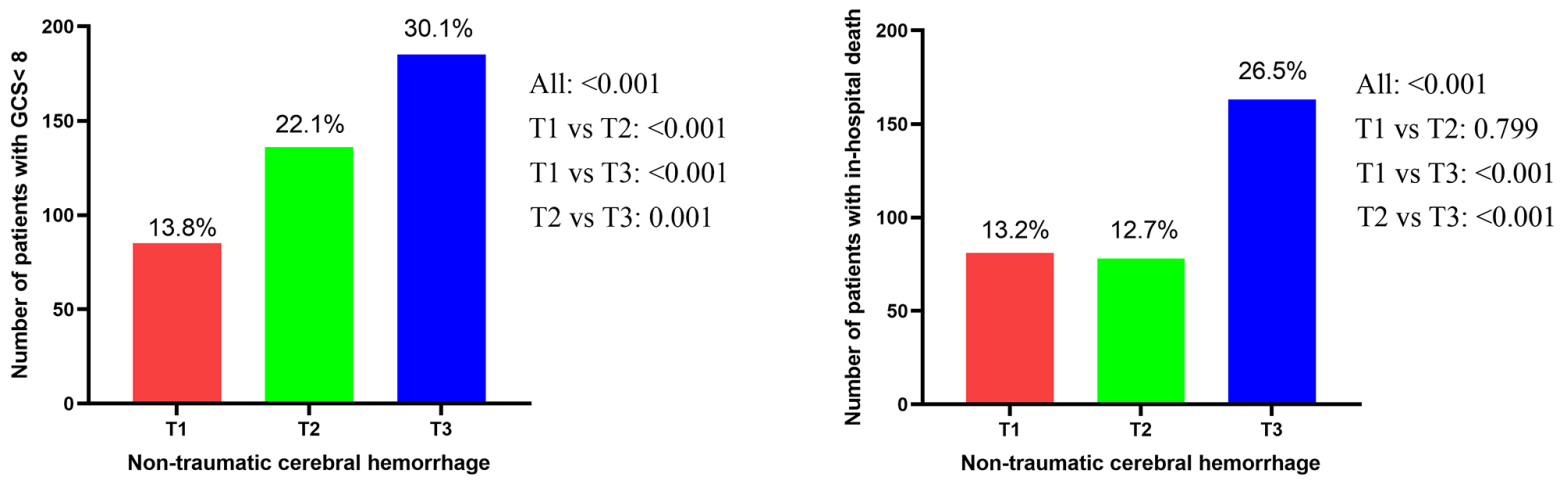
The incidence of severe consciousness disturbance and in-hospital death among three groups according to tertiles of CV in the non-traumatic cerebral hemorrhage patients

**Fig. 3:**
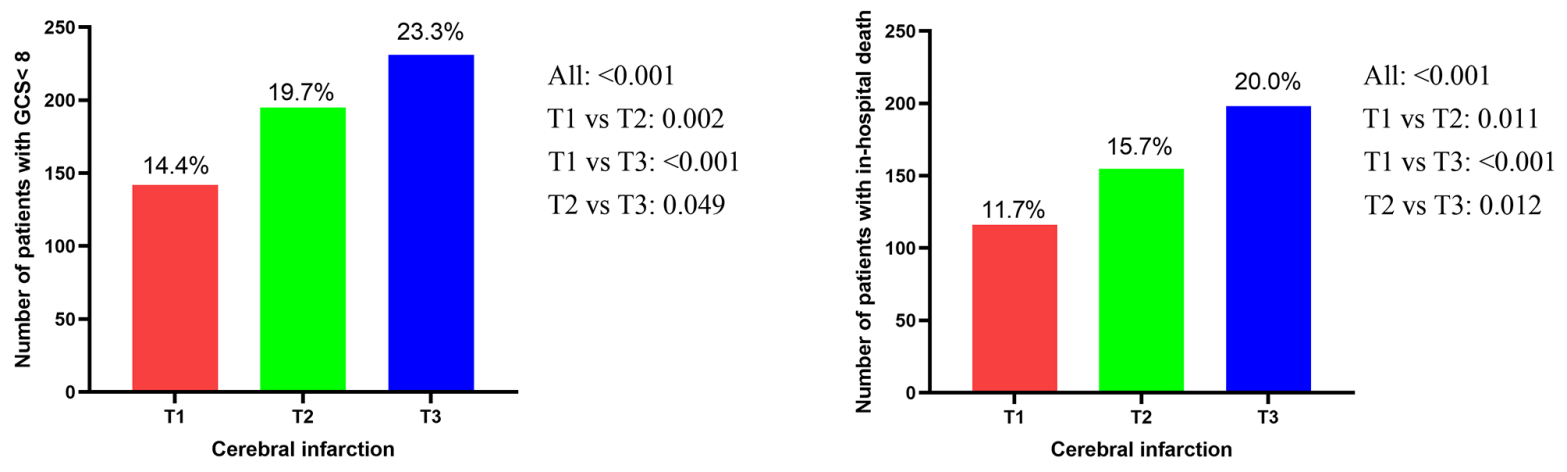
The incidence of severe consciousness disturbance and in-hospital death among three groups according to tertiles of CV in the cerebral infarction patients

In the present study, patients were stratified into three groups by the tertiles of CV in the non-traumatic hemorrhage cohort (< 14.1%, 14.1–21.5%, > 21.5%) and cerebral infarction cohort (< 16.0%, 16.0-24.7%, > 24.7%) according to the glycemic variability. In the non-traumatic cerebral hemorrhage cohort, the incidence of severe disturbance of consciousness and in-hospital death were 13.8% and 13.2% in patients with lower glycemic variability (CV < 14.1%), 22.1% and 12.7% in those with medium glycemic variability (CV 14.1–21.5%), 30.1% and 26.5% in those with higher glycemic variability (CV > 21.5%), respectively (Fig. 2). In the cerebral infarction, the similar results were shown in the Fig. 3.

The Cox hazard regression model was applied to reduce the effect of the covariates on the outcome. (Model 1: unadjusted; Model 2: adjusted for age, sex, race, and BMI; Model 3: adjusted for age, sex, race, BMI, CAD, diabetes, heart failure, hypertension, sepsis, AF, MV. CRRT, antidiabetes therapy, antihypertensive therapy, and glucose measurement number.)

Cox hazard regression analysis showed that log-transformed CV was significantly associated with severe cognitive impairment and in-hospital death. The relevant analysis of patients with non-traumatic cerebral hemorrhage presented that in unadjusted (HR [95% CI], 1.864 (1.578–2.201)), partially adjusted (HR [95% CI], 1.830 (1.547–2.165)), and fully adjusted (HR [95% CI], 1.394 (1.143–1.699)) models, log-transformed CV was an independent risk factor of severe cognitive impairment. Furthermore, the results indicated that a higher CV was also significantly related to the patients’ in-hospital mortality. In unadjusted (HR [95% CI], 1.927 (1.600-2.322)), partially adjusted (HR [95% CI], 1.876 (1.552–2.267)), and fully adjusted (HR [95% CI], 1.812 (1.452–2.260)) models (Table 3).

**Table 3:** Cox hazard regression for the association of glycemic variability with severe consciousness disturbance and in-hospital death

Outcomes	HR Per unit of Log CV	P value	HR Per unit of Log CV	P value	HR Per unit of Log CV	P value
	Model 1		Model 2		Model 3	
Non-traumatic cerebral hemorrhage						
GCS < 8	1.864 (1.578–2.201)	< 0.001	1.830 (1.547–2.165)	< 0.001	1.394 (1.143–1.699)	0.001
In-hospital death	1.927 (1.600-2.322)	< 0.001	1.876 (1.552–2.267)	< 0.001	1.812 (1.452–2.260)	< 0.001
Both	1.914 (1.660–2.206)	< 0.001	1.872 (1.622–2.161)	< 0.001	1.560 (1.318–1.845)	< 0.001
Cerebral infarction						
GCS < 8	1.486 (1.285–1.719)	< 0.001	1.486 (1.285–1.718)	< 0.001	1.215 (1.032–1.432)	0.020
In-hospital death	1.545 (1.316–1.814)	< 0.001	1.500 (1.277–1.760)	< 0.001	1.234 (1.030–1.477)	0.022
Both	1.566 (1.387–1.768)	< 0.001	1.551 (1.374–1.751)	< 0.001	1.275 (1.112–1.462)	< 0.001

Abbreviations: HR, Hazard ratio; GCS, Glasgow coma scale; BMI, body mass index; CAD, coronary artery disease; AF, atrial fibrillation; MV, mechanical ventilation; CRRT, continuous renal replacement therapy

Model 1: unadjusted;

Model 2: adjusted for age, sex, race, and BMI;

Model 3: adjusted for age, sex, race, BMI, CAD, diabetes, heart failure, hypertension, sepsis, AF, MV. CRRT, antidiabetes therapy, antihypertensive therapy, and glucose measurement number

Analysis of patients with cerebral infarction also demonstrated that the log-transformed CV was an independent risk factor of severe disturbance of consciousness (unadjusted HR, [95% CI] 1.486 (1.285–1.719); partially adjusted HR, [95% CI] 1.486 (1.285–1.718); fully adjusted HR, [95% CI] 1.215 (1.032–1.432)). Additionally, compared with patients in the lowest tertile, patients in the higher tertile of CV was significantly related to higher risk of all-cause in-hospital death (unadjusted HR, [95% CI] 1.545 (1.316–1.814); partial adjusted HR, [95% CI] 1.500 (1.277–1.760); fully adjusted HR, [95% CI] 1.234 (1.030–1.477)) (Table 3).

**Fig. 4:**
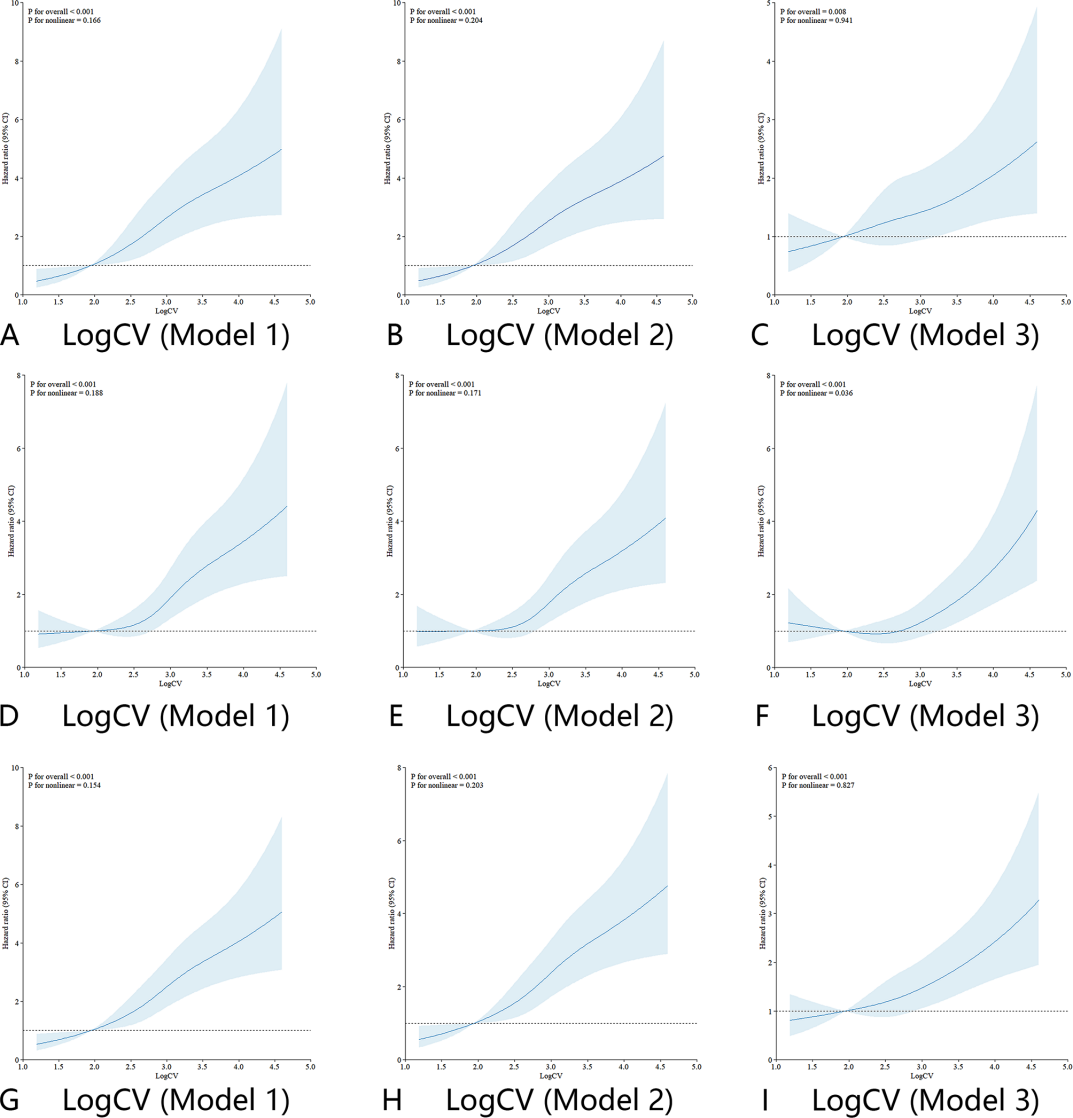
RCS curve of LogCV and HR in patients with non-traumatic cerebral hemorrhage: (**A**, **B**, and **C**) RCS curve for severe disturbance of consciousness. (**D**, **E**, and **F**) RCS curve for hospital mortality. (**G**, **H**, and **I**) RCS curve for both of severe disturbance of consciousness and hospital death

Then, restricted cubic splines (RCS) was used to evaluate possible nonlinear association of the LogCV with outcomes. The results of RCS demonstrated nearly linearity association between LogCV and outcomes (p for non-linearity > 0.05). In the patients diagnosed with non-traumatic cerebral hemorrhage, the risk of severe cognitive impairment showed approximately linear association with LogCV (Model 1: 0.166; Model 2: 0.204; Model 3: 0.941). The increasing LogCV increased approximately linearly with the risk of patients’ in-hospital mortality (Model 1: 0.188; Model 2: 0.171) (Fig. 4). In patients with cerebral infarction, LogCV also demonstrated approximately linearly with the risk of severe cognitive impairment (Model 1: 0.089; Model 2: 0.069; Model 3: 0.795) and all-cause in-hospital death (Model 1:0.292; Model 2: 0.278; Model 3:0.665) (Additional file, Figure S1).

### Sensitivity analyses and correlation analyses

**Fig. 5:**
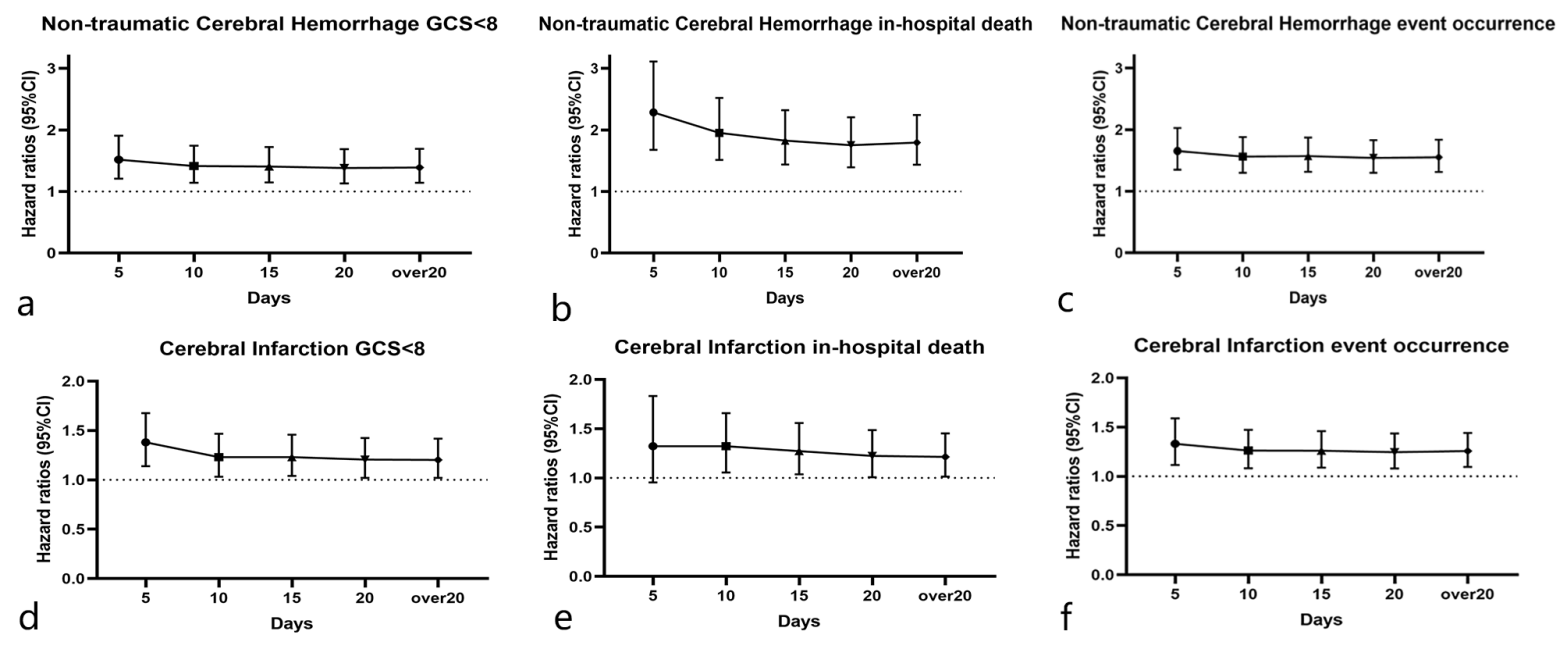
Sensitivity analyses of glycemic variability on outcomes in the different time. Adjustment was made for age, sex, race, BMI, CAD, diabetes, heart failure, hypertension, sepsis, AF, MV. CRRT, antidiabetes therapy, antihypertensive therapy, and glucose measurement number. (**a-c**): glycemic variability on cognitive impairment, in-hospital death, and both of outcomes in the non-traumatic hemorrhage patients; (**d-f**): glycemic variability on cognitive impairment, in-hospital death, and both of outcomes in the cerebral infarction patients

Sensitivity analyses were performed to elucidate the effect of glycemic variability and exclude reverse causality in different time windows. The results showed that there was no significant difference of the effect of glycemic variability in different time windows (Fig. 5). According to the results of the Pearson correlation coefficient test in the non-traumatic cerebral hemorrhage patients group, a direct and statistically significant correlation was observed between glycemic variability on number of glycemic measurements (*r* = 0.222, *p* < 0.001), and length of hospital stay (*r* = 0.141, *p* < 0.001). In the cerebral infarction patients, a statistically significant correlation was also observed between glycemic variability on number of glycemic measurements (*r* = 0.205, *p* < 0.001), and length of hospital stay (*r* = 0.177, *p* < 0.001) (Additional file, Figure S2).

### Subgroup analyses stratified by glycemic variability

**Fig. 6:**
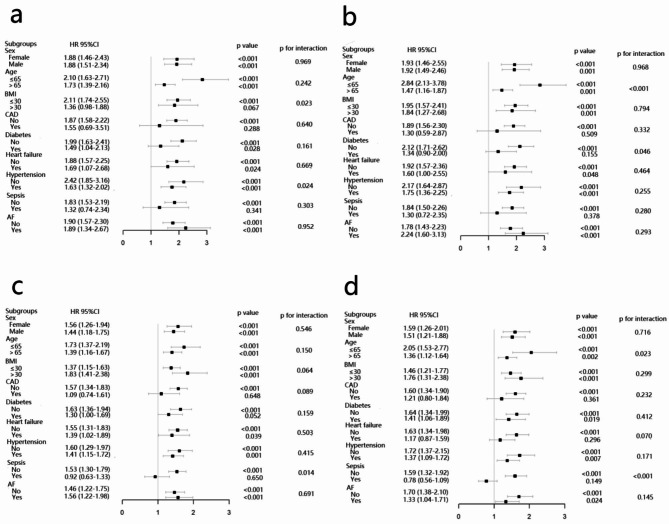
Subgroup analyses for the association of LogCV with endpoints in the non-traumatic cerebral hemorrhage and cerebral infarction patients. (**a**) LogCV and severe disturbance of consciousness in the non-traumatic cerebral hemorrhage patients. (**b**) LogCV and in-hospital death in the non-traumatic cerebral hemorrhage patients. (**c**) LogCV and severe disturbance of consciousness in the cerebral infarction patients. (**d**) LogCV and in-hospital death in the cerebral infarction patients

Further assessment of the risk stratification value of the natural log-transformed CV for outcomes measure was performed in various subgroups of the study population, including sex, age, BMI, CAD, diabetes mellitus, heart failure, hypertension, sepsis and atrial fibrillation. In the non-traumatic cerebral hemorrhage cohort, increased LogCV was significantly related to higher risk of severe consciousness disturbance in various subgroups, including sex (male or female), age (> 65 or ≤ 65 years), BMI (BMI ≤ 30 kg/m^2^), CAD (without), sepsis (without), and with or without diabetes mellitus, heart failure, atrial fibrillation, hypertension. Interestingly, it seemed that the LogCV was more prominent in patients with BMI ≤ 30 kg/m^2^ [HR (95% CI) BMI ≤ 30 kg/m^2^ 2.11 (1.74–2.55) vs. BMI>30 kg/m^2^ 1.36 (0.98–1.88) p for interaction = 0.023] and without hypertension [HR (95% CI) without hypertension 2.42 (1.85–3.16) vs. with hypertension 1.63 (1.32–2.02) p for interaction = 0.024] (Fig. 6a). Similar results were yielded in the stratified analyses of the LogCV and in-hospital mortality (Fig. 6b). We also investigated the association between the LogCV and outcome in the cerebral infarction cohort, which had been shown in the Fig. 6c and d.

## Discussion

In the present retrospective study derived from the MIMIC-IV database, we found that LogCV, an indicator of glycemic variability, was related to an elevated risk of severe consciousness disturbance and greater in-hospital all-cause mortality rate in critically ill patients with CVD. Our results also demonstrated that the LogCV was approxiamately linearly associated with severe cognitive decline and in-hospital death.

Despite rapid improvements in early diagnosis and timely intervention, CVD remains the leading cause of mortality and disability in global [26]. Diabetes mellitus is generally considered a risk factor for CVD [27, 28], and is strongly related to the incidence of ischemic and hemorrhagic strokes [29]. High glycemic variability has also been implicated in the risk of composite vascular events [30]. Previous studies performed only in the general population demonstrated the relationship between glycemic variability and cognitive decline. A large cohort study indicated that glycemic variability was associated with an increased risk of long-term cognitive decline [31]. In young people with diabetes, abnormal glycemic variability had a negative effect on cognition [32]. The abnormality of blood glucose metabolism in ICU patients requires attention, as it differs from that of the general population. Abnormal blood glucose levels are very prevalent among critically ill patients, and various stressors can trigger the blood glucose disorders of ICU patients through regulating multiple hormones (including glucagon, cortisol, thyroxine and growth hormone) to meet organs’ energy need; however, disorders of blood glucose also have a negative impact [33]. In the ICU, artificial nutritional support from enteral and parenteral nutrition resulted in an elevated possibility of blood glucose disorders [34].

Previous study had demonstrated that fluctuating glucose levels were more deleterious to neuron cell functioning compared to consistently low or high levels [35]. Rawlings et al. found that glycemic variability led to an increased risk of dementia and cognitive decline [31]. In one human experimental research, Rizzo et al. have shown that an impairment of cognitive functioning was related to daily glycemic variability by continuous subcutaneous glucose monitoring [36].

Several scholars have suggested that high glycemic variability can have more detrimental effect on prognosis than constant high blood glucose. High glycemic variability is significantly associated with an increase in mortality, even when blood glucose levels are well under control, suggesting that blood glucose fluctuation could be used as a predictor of residual risk of death in diabetic patients with well-controlled glucose level [37, 38]. Ma et al. also indicated that hypoglycemia and glycemic variability played a role in all-cause mortality in ICU patients instead of hyperglycemia [39]. In patients with cardiovascular disease, patients with high glycemic variability had higher risk of developing major adverse cardiovascular events and all-cause mortality [40, 41]. In the present study, we found that the effect of glycemic variability on all-cause in-hospital was partly mediated by severe consciousness disturbance. High blood glucose fluctuation could influence several organs, and increase the incidence of cardiovascular events including heart failure, ventricular arrhythmias, myocardial infarction, as well as renal dysfunction, immunity disorders and nerve damage [10, 42]. The increased in-hospital mortality rate might be elucidated by the above potential mechanism.

Another finding from subgroup analysis suggested that elevated glycemic variability was more likely to significantly increase the risk of poor outcome in critical patients with non-traumatic hemorrhage whose BMI < 30 kg/m^2^, which was consistent with previous study [43]. The possible mechanism might be patients with high BMI were expected to be more resilient to the deleterious effect [44]. As for the patients without hypertension, previous study also indicated that anti-oxidative stress marker was more significantly decreased the risk of poor outcome in patients without hypertension [45].

In our findings, monitoring the glycemic variability can play a role in reducing the severe consciousness decline and in-hospital death for critically ill patients with CVD. Similarly, a series of brand-new nutrition formulae for ICU patients were applied to reduce glycemic variability. A meta-analysis of randomized-controlled trials was dedicated to develop a new glycemic-control formulae to better improve glycemic control [46]. Additionally, one observational study proposed enteral formulae consisting of sustained-release starch, and found it could significantly improve glycemic stability in patients with severe acute pancreatitis and stress hyperglycemia compared to the control cohort [47].

The promising findings of this retrospective study call for more careful management of blood glucose and severe consciousness disturbance in ICU patients with CVD. Due to its association with prognosis, the stability of glycemic variability requires essential attention. Additionally, our study revealed the significance of glycemic variability on the severity of consciousness disturbance and prognosis of patients with CVD, which helps physicians pay more attention to detecting consciousness change and providing timely treatment, such as medication, correction of homeostasis disorders. The relevant medical interventions contribute to reducing the in-hospital mortality related abnormal blood glucose fluctuation.

There were several limitations should be mentioned in the present study. Firstly, blood glucose measurements were not standardized for each patient and were not continuous, different therapy and food intake may also affect the frequency of blood glucose measurements. Secondly, due to the limitations of MIMIC-IV database, some potential confounders were not included, such as dietary patterns, physical activity.

## Conclusion

Glycemic variability was significantly associated with an increased risk of consciousness disturbance and in-hospital death in critically ill patients with cerebrovascular disease. Therefore, dynamic monitoring of blood glucose may be beneficial in assessing the risk and predicting the prognosis of this patient population.

### Electronic supplementary material

Below is the link to the electronic supplementary material.

**Additional file 1**: Figure S1. RCS curve of LogCV and HR in patients with cerebral infarction: (A, B, and C) RCS curve for severe disturbance of consciousness. (D, E, and F) RCS curve for hospital mortality. (G, H, and I) RCS curve for both of severe disturbance of consciousness and hospital death.

**Additional file 2**: Figure S2: The relationship between glycemic variability and number of glycemic measurement and length of hospital stay. (a) the relationship between glycemic variability and number of glycemic measurement in the non-traumatic cerebral hemorrhage group; (b) the relationship between glycemic variability and number of glycemic measurement in the cerebral infarction group; (c) the relationship between glycemic variability and length of hospital stay in the non-traumatic cerebral hemorrhage group; (d) the relationship between glycemic variability and length of hospital stay in the cerebral infarction group.

## Data Availability

The datasets generated and analyzed during the current study are available from the corresponding author on reasonable request.

## Abbreviations

CVD: cerebrovascular disease
MIMIC-IV: Medical Information Mart for Intensive Care IV
CV: coefficient of variation
SD: standard deviation
ICU: intensive care unit
BIDMC: Beth Israel Deaconess Medical Center
SQL: Structured Query Language
CAD: coronary artery disease
COPD: chronic obstructive pulmonary disease
AF: atrial fibrillation
WBC: white blood cell
RBC: red blood cell
PT: prothrombin time
PTT: active partial thromboplastin time
SOFA: Sequential Organ Failure Assessment Score
SAPSII: Simplified Acute Physiology Score
APSIII: Acute Physiology Score III
OASIS: Oxford Acute Severity of Illness Score
GCS: Glasgow coma scale
OR: odds ratio
CI: confidence interval
BP: blood pressure
BMI: body mass index
MV: mechanical ventilation
CRRT: continuous renal replacement therapy
RCS: restricted cubic spline
