# Supplementary figures and images for: Association of glycemic variability with death and severe consciousness disturbance among critically ill patients with cerebrovascular disease: analysis of the MIMIC-IV database
Source: Cardiovasc Diabetol. 2023 Nov 16;22:315. doi: 10.1186/s12933-023-02048-3 (PMC10652479; doi:10.1186/s12933-023-02048-3)

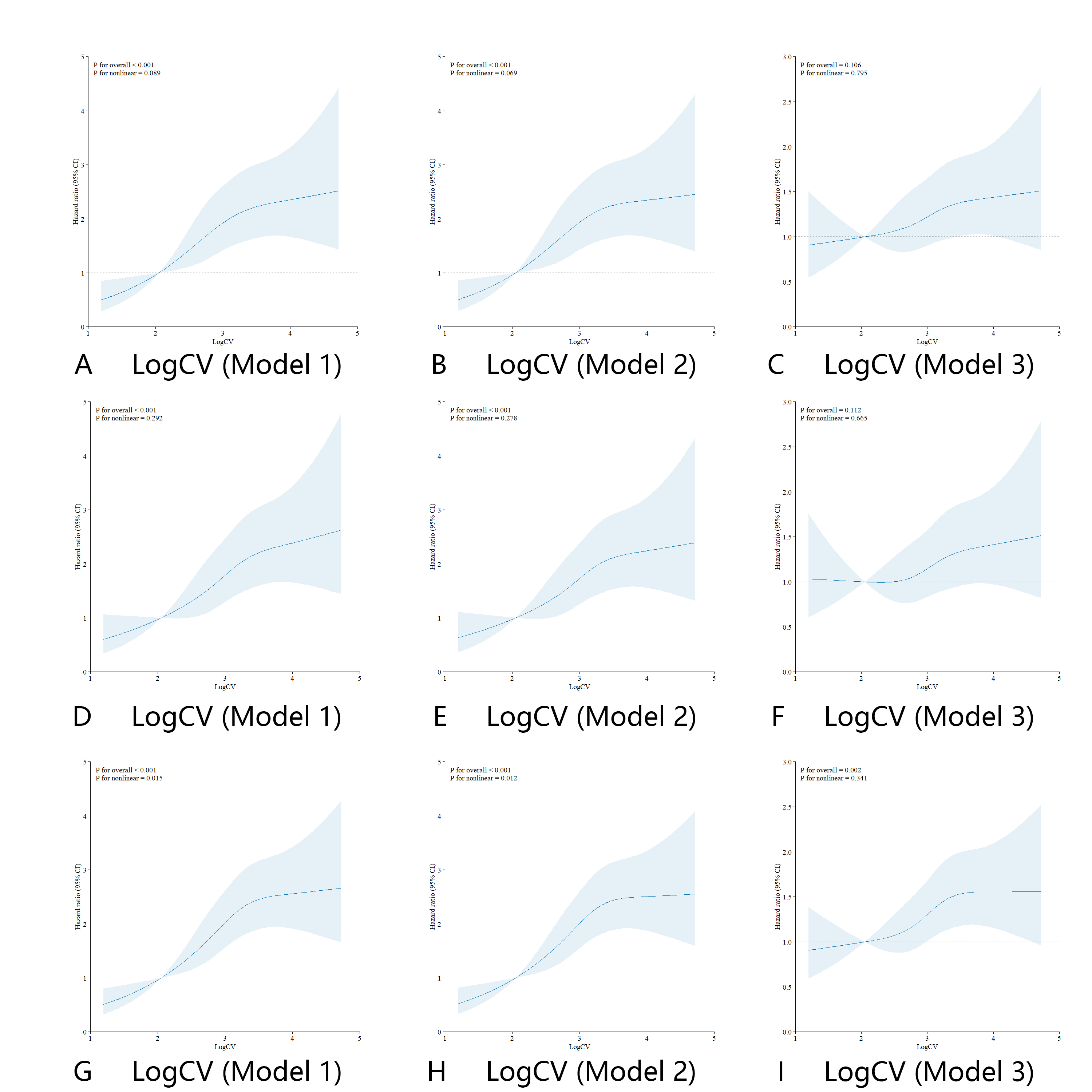

Supplement: Supplementary file 1 — Additional file 1: Figure S1. RCS curve of LogCV and HR in patients with cerebral infarction: (A, B, and C) RCS curve for severe disturbance of consciousness. (D, E, and F) RCS curve for hospital mortality. (G, H, and I) RCS curve for both of severe disturbance of consciousness and hospital death. [file 12933_2023_2048_MOESM1_ESM.png]

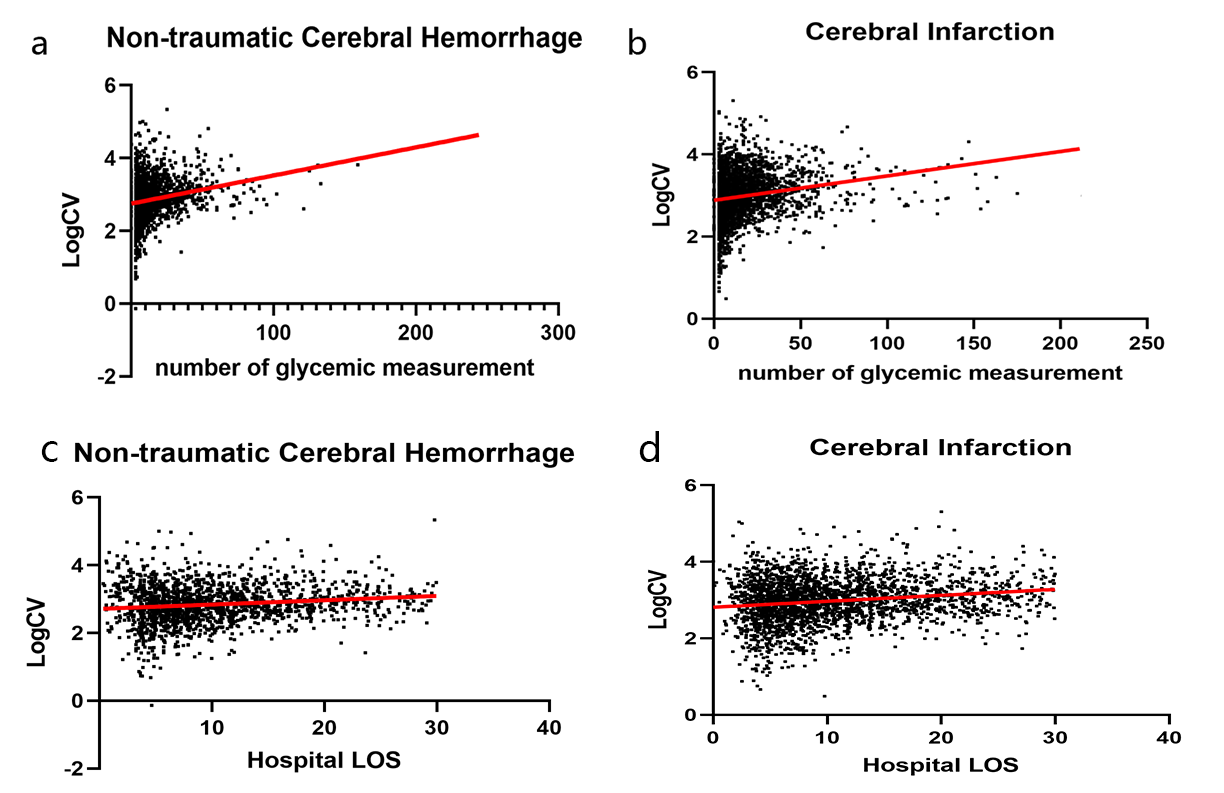

Supplement: Supplementary file 2 — Additional file 2: Figure S2: The relationship between glycemic variability and number of glycemic measurement and length of hospital stay. (a) the relationship between glycemic variability and number of glycemic measurement in the non-traumatic cerebral hemorrhage group; (b) the relationship between glycemic variability and number of glycemic measurement in the cerebral infarction group; (c) the relationship between glycemic variability and length of hospital stay in the non-traumatic cerebral hemorrhage group; (d) the relationship between glycemic variability and length of hospital stay in the cerebral infarction group. [file 12933_2023_2048_MOESM2_ESM.png]
